# Macrogenomics-based analysis of rumen microbial composition and their metabolic pathways in yaks under different dietary concentrate-to-forage ratios

**DOI:** 10.3389/fmicb.2025.1587474

**Published:** 2025-08-06

**Authors:** Fajie Gou, Yincang Han, Yonggang Sun, Weiqing Ding, Shenwei Jin, Yaqian Liu, Jianyu Chen

**Affiliations:** ^1^Academy of Animal Science and Veterinary Medicine, Qinghai University, Xining, China; ^2^Key Laboratory of Plateau Livestock Genetic Resources Protection and Innovative Utilization of Qinghai Provincial, Xining, China

**Keywords:** macrogenomics, concentrate-to-forage ratios, yak, rumen microorganisms, microbial metabolic pathways

## Abstract

This study aimed to investigate the regulatory mechanisms underlying feed efficiency (FE) in yaks by analyzing the composition of rumen microorganisms and their major metabolic pathways using metagenomic analysis under different dietary concentrate-to-forage ratios. A total of 40 Qinghai Plateau yaks (8–9 months old) with similar body weights (68.725 ± 18.973 kg) were randomly assigned to four treatment groups (*n* = 10per group). The experimental groups were fed diets with concentrate-to-forage ratios of 80:20 (C80), 65:35 (C65), 50:50 (C50), and 35:65 (C35), respectively. The study included a 15-day pre-feeding period followed by a 105-day experimental feeding period. The results indicated that the total weight gain in the C65 group was significantly higher than in the C50 and C35 groups by 29.91 and 28.97%, respectively (*p* < 0.05). Additionally, the rumen pH in the C80 group was significantly higher than in the C65, C50, and C35 groups (*p* < 0.05). Metagenomic analysis revealed significant differences (*p* < 0.05) in bacterial and archaeal community compositions across groups. Bacteroidota, Bacillota, Prevotella, Bacteroides, and Ruminococcus were identified as the dominant bacterial taxa at the phylum and genus levels. Functional analysis of rumen microbial metabolism showed that in the C35 group, pathways related to starch and sucrose metabolism, as well as fructose and mannose metabolism, were significantly different from those in other groups. The C35 group exhibited higher activity in functional pathways related to starch and sucrose metabolism, fructose and mannose metabolism, cellulose degradation, and methanolysis. In contrast, the C80 group showed greater activity in cellulose degradation and methane metabolism. Notably, the C65 group exhibited the highest activity in sugar metabolism pathways (ko00500), facilitating starch and soluble sugar degradation and the rapid conversion of pyruvic acid into acetic acid and propionate. This enhanced energy utilization efficiency, suggesting a superior capacity for sugar metabolism. In conclusion, the dietary composition of the C65 group demonstrated the most favorable effects on growth performance, rumen fermentation optimization, and microbial balance maintenance.

## 1 Introduction

The yak (*Bos grunniens*) is a domesticated ruminant endemic to the Tibetan Plateau and its surrounding regions, playing a crucial role in the livelihoods and production systems of local herders due to its exceptional cold tolerance and efficient utilization of roughage (Hu et al., 2019). Yaks primarily subsist on natural pastures rich in crude fiber, and their digestive system—particularly the rumen—has evolved a specialized microbial community adapted to high-fiber diets. As the primary site of fermentation in ruminants, the rumen harbors a diverse microbial ecosystem, including bacteria, archaea, fungi, and protozoa. These microorganisms engage in synergistic interactions to degrade plant fibers, producing volatile fatty acids (VFAs) through fermentation, which serve as the host’s primary energy source (Hua et al., 2022; Sirohi et al., 2012; Yue et al., 2013).

In yak husbandry, the dietary concentrate-to-forage ratio (C:F) is a key determinant of rumen microbial structure and function. Traditional yak farming relies on natural grazing, with a diet predominantly composed of high-fiber forage. However, in modern intensive farming systems, an increased proportion of concentrate feed (corn, barley, soybean meal) is often incorporated to enhance growth rates and production performance (Jiang et al., 2022). Adjustments in C:F ratios significantly impact the rumen environment, influencing factors such as pH, redox status, and ammoniacal nitrogen content, which in turn affect the composition and metabolic activity of rumen microorganisms. Studies have shown that a higher proportion of concentrate feed increases the availability of fermentable carbohydrates in the rumen, promoting the proliferation of starch-degrading bacteria (*Streptococcus bovis*, Prevotella spp.). However, this dietary shift may concurrently inhibit the growth of fiber-degrading bacteria (*Fibrobacter succinogenes*, *Ruminococcus flavefaciens*), ultimately reducing the efficiency of cellulose and hemicellulose degradation (Palmonari et al., 2024; Pang et al., 2022a,b). Additionally, high-concentrate diets can lead to a decline in rumen pH, disrupting the microbial equilibrium and predisposing yaks to rumen acidosis, which negatively affects both health and production efficiency (Elmhadi et al., 2022; Zebeli et al., 2012). Therefore, optimizing the dietary C:F ratio to maintain microbial diversity and functional stability in the rumen has become a pressing scientific challenge in yak nutrition and management.

With advancements in high-throughput sequencing technology, metagenomics has emerged as a powerful tool for investigating rumen microbial communities. Compared to traditional 16SrRNA sequencing, metagenomics provides a more comprehensive understanding of the genetic composition, functional potential, and metabolic pathways of rumen microorganisms, thereby enabling a deeper exploration of microbial functions and their interactions with the host (Kelleci and Comlekcioglu, 2016). However, research on the effects of different C:F levels on yak rumen microbiota—particularly in terms of microbial adaptation to varying dietary conditions—remains relatively limited. To address this gap, the present study employed metagenomic techniques to analyze the effects of different C:F ratios on the structure, functional gene expression, and metabolic pathways of the yak rumen microbiome. This study aims to elucidate the synergistic interactions between the host and its rumen microbes, providing novel insights into the dietary adaptability of yaks. Furthermore, the findings offer a theoretical foundation for optimizing feeding strategies, improving feed efficiency, and maintaining rumen microbial homeostasis in yak production systems.

## 2 Materials and methods

### 2.1 Animal ethics statement

The use of animals and all experimental protocols were authorized by the Institutional Animal Care and Use Committee of Qinghai University (Xining, China).

### 2.2 Laboratory animals and design

The present study was conducted from January to May 2024 at the Bianma Meron Palm Cooperative in Qilian County, Haibei Tibetan Autonomous Prefecture, Qinghai Province, China. Forty Qinghai Plateau-type yaks, aged 8 to 9 months and in good health with similar body weights (68.725 ± 18.973 kg), were selected and randomly assigned to four groups, with 10 replicates per group. The four treatment groups were fed a total mixed ration (TMR) with concentrate-to-roughage ratios of 80:20 (C80 group), 65:35 (C65 group), 50:50 (C50 group), and 35:65 (C35 group), respectively. The experimental diets were formulated according to the Chinese Standard for Beef Cattle Feeding (NY/T 815–2004), with the composition and nutrient content outlined in Table 1. Prior to the start of the experiment, the pens were cleaned and disinfected. The yaks were then grouped and ear-tagged for identification. Feeding occurred twice daily, at 09:00 and 17:00, ensuring that some feed was left over after each feeding. Leftover feed was weighed before the morning feeding the following day. Throughout the experimental period, the yaks had free access to both feed and water.

**Table 1:** Composition and nutrient level of experimental feed (dry matter basis).

Items	Groups			
	C80	C65	C50	C35
Ingredients				
Oat hay (%)	20	35	50	65
Corn (%)	36.5	27.5	16.7	6.5
Wheat (%)	9	6.8	5.7	4.3
Wheat bran (%)	8.5	8	7.3	7
Soybean meal (%)	7.6	5.5	5	3.5
Rapeseed meal (%)	9	8.2	7.3	6.7
Cottonseed meal (%)	6.4	6	5	4
premix^1^ (%)	2	2	2	2
NaCl (%)	1	1	1	1
Total	100	100	100	100
Nutrient level				
Metabolizable energy (ME/(MJ/kg)^2^)	9.9	9.35	8.4	8.1
Dry matter (%)	83.0	83.0	80.3	75.8
Crude protein (%)	13.081	13.02	13.0	13.03
Neutral detergent fiber (%)	61.5	60.3	54.1	59.2
Acid detergent fiber (%)	37.06	37.8	34.3	29.9
Crude fat (g/kg)	15.0	22.0	25.0	14.0
Ca (mg/kg)	286.0	507.0	524.0	396.0
P (mg/kg)	115.79	261.25	248.36	168.58

(1) Each kg of premix contains Co 0.20 mg, Cu 15 mg, I 0.80 mg, Fe 100 mg, Mn 25 mg, Se 0.17 mg, Zn 50 mg, VA 3000 IU, VD 350 IU, VE 40 IU. (2) The metabolic energy of nutrient level was calculated, and the rest was measured.

### 2.3 Sample collection and indicator measurement

#### 2.3.1 Measurement of growth performance

Yaks were weighed prior to the morning feeding at both the beginning and end of the trial to determine total weight gain and average daily gain (ADG) over the course of the study. In addition, body height, body slant length, and chest circumference were measured at the start and conclusion of the trial to assess growth and development.

#### 2.3.2 Collection of rumen fluid

Six yaks from each group were randomly selected and slaughtered after a 24-h fasting period, with both feed and water withheld. The rumen, reticulum, omasum, abomasum, and duodenum were carefully separated using sutures to prevent chyme reflux between adjacent compartments. Rumen fluid was then collected by first discarding an initial sample, followed by the extraction of 80 mL of rumen fluid. The pH was measured immediately after filtration through four layers of degreased gauze using a portable pH meter (PHS-3C, Leijun Technology Co.).

#### 2.3.3 Determination of rumen fermentation parameters

The rumen fermentation parameters mainly included pH, NH3-N concentration, BCP concentration and total VFA concentration (acetic acid, propionic acid, isobutyric acid, butyric acid, valeric acid, isovaleric acid, hexanoic acid and heptanoic acid). The pH of rumen fluid was determined by a PHB-4 portable pH meter; the ammonia-nitrogen (NH3-N) content was determined by a blood ammonia kit, which was ordered from Nanjing Chengjian Bioengineering Research Institute, and the test procedure was described in the kit manual; the volatile fatty acid (VFA) content was determined by a gas chromatograph using an AgilentTechnologies6890N-GC; and the total VFA concentration (acetic acid, propionic acid, butyric acid, valeric acid, isovaleric acid, hexanoic acid, and heptanoic acid) was determined by a gas chromatograph. The content of bacterial body protein (BCP) was determined by spectrophotometer. The remaining rumen fluid was dispensed into 10 mL centrifuge tubes and stored at −20°C for measurement.

#### 2.3.4 DNA extraction, library construction, and metagenomic sequencing

Total genomic DNA was extracted from rumen fluid samples using the Mag-Bind® DNA Kit (Omega Bio-tek, Norcross, GA, United States) according to manufacturer’s instructions. Concentration and purity of extracted DNA was determined with TBS-380 and NanoDrop2000, respectively. DNA extract quality was checked on 1% agarose gel.

DNA extract was fragmented to an average size of about 350 bp using Covaris M220 (Gene Company Limited, China) for paired-end library construction. Paired-end library was constructed using NEXTFLEX® Rapid DNA-Seq (Bioo Scientific, Austin, TX, United States). Adapters containing the full complement of sequencing primer hybridization sites were ligated to the blunt-end of fragments. Paired-end sequencing was performed on Illumina NovaSeq (Illumina Inc., San Diego, CA, United States) at Majorbio Bio-Pharm Technology Co., Ltd. (Shanghai, China) using NovaSeq 6,000 S4 Reagent Kit v1.5 (300 cycles) according to the manufacturer’s instructions.^1^

#### 2.3.5 Sequence quality control and genome assembly

The data were analyzed on the free online platform of Majorbio Cloud Platform^2^. Briefly, the paired-end Illumina reads were trimmed of adaptors, and low-quality reads (length<50 bp or with a quality value <20) were removed by fastp (Chen et al., 2018) (https://github.com/OpenGene/fastp, version 0.23.0). Metagenomics data were assembled using MEGAHIT (Li et al., 2015) (https://github.com/voutcn/megahit, version 1.1.2), contigs with a length ≥ 300 bp were selected as the final assembling result, and then the contigs were used for further gene prediction and annotation.

#### 2.3.6 Gene prediction, taxonomy, and functional annotation

Open reading frames (ORFs) from each assembled contig were predicted using Prodigal/MetaGene^3^ (Hyatt et al., 2010; Noguchi et al., 2006). The predicted ORFs with a length ≥ 100 bp were retrieved and translated into amino acid sequences using the NCBI translation table^4^.

A non-redundant gene catalog was constructed using CD-HIT (Fu et al., 2012) (http://www.bioinformatics.org/cd-hit/, version 4.6.1) with 90% sequence identity and 90% coverage. High-quality reads were aligned to the non-redundant gene catalogs to calculate gene abundance with 95% identity using SOAPaligner (Li et al., 2008) (https://academic.oup.com/bioinformatics/article/24/5/713/203564).

### 2.4 Statistical analysis

Data analysis in this study was performed using R software (version 3.3.3) and SPSS (version 27.0), and all data were expressed as mean ± standard deviation. Data were analyzed by one-way ANOVA (One-way ANOVA) if they conformed to a normal distribution, and differences between groups were analyzed using Duncan’s Multiple Range Test (Duncan’s Multiple Comparisons). All statistical tests were two-sided, with *p* < 0.05 indicating significant differences and *p* < 0.01 indicating highly significant differences. Data visualization was performed using R ggplot2, GraphPad Prism.

## 3 Results and analysis

### 3.1 Effect of dietary concentrate/crude ratio on growth performance of yak cattle

As shown in Table 2, at the end of the experiment, the body slant length of yaks in the C65 group was significantly greater than that of yaks in the C50 group (*p* < 0.05). The total weight gain of yaks in the C65 group was also significantly higher than that of yaks in the C50 and C35 groups, by 29.91 and 28.97%, respectively (*p* < 0.05). Additionally, the average daily weight gain of yaks in the C65 group was 17.74% higher than that of yaks in the C35 and C50 groups (*p* < 0.05). No significant differences were observed in other growth performance parameters (*p* > 0.05).

**Table 2:** Effects of dietary concentrate/crude ratios on growth performance of yaks (*Bos grunniens*).

Groups	C80	C65	C50	C35	*P*-value
Starting weight (kg)	71.60 ± 4.47	73.50 ± 3.48	70.30 ± 3.05	72.20 ± 2.81	0.199
Initial height (cm)	78.60 ± 5.68	79.40 ± 2.51	79.60 ± 4.39	78.40 ± 4.51	0.965
Initial Bust (cm)	111.20 ± 7.09	109.80 ± 6.26	111.80 ± 1.92	115.20 ± 3.42	0.417
Initial body slant length (cm)	92.00 ± 4.12^b^	94.20 ± 3.63^ab^	99.80 ± 7.89^a^	92.40 ± 1.67^b^	0.039
Closing weight (kg)	141.40 ± 22.71	148.40 ± 11.19	122.80 ± 26.34	125.40 ± 21.03	0.129
End body height (cm)	97.80 ± 5.22	98.60 ± 3.58	96.80 ± 5.45	96.20 ± 9.73	0.936
End Bust (cm)	157.80 ± 7.76	151.60 ± 4.45	151.20 ± 8.16	156.00 ± 6.74	0.375
End body is obliquely long (cm)	117.80 ± 8.41^ab^	122.00 ± 4.53^a^	110.00 ± 8.45^b^	112.80 ± 7.05^ab^	0.019
Total weight gain (kg)	69.80 ± 1.27^ab^	74.90 ± 5.61^a^	52.50 ± 3.77^b^	53.20 ± 2.28^b^	0.046
Average daily weight gain (kg)	0.58 ± 0.08	0.62 ± 0.03	0.51 ± 0.11	0.51 ± 0.07	0.119

Peer data with the same lowercase letter indicates a non-significant difference (*P* > 0.05), and a different letter indicates a significant difference (*P* < 0.05), as shown in the table below.

### 3.2 Effect of dietary concentrate/crude ratio on rumen fermentation parameters in yak (*Bos grunniens*)

As shown in Table 3, the rumen pH of yaks in the C80 group was significantly higher than that of yaks in the C65, C50, and C35 groups (*p* < 0.05). No significant differences in rumen pH were observed among the C65, C50, and C35 groups (*p* > 0.05). The NH₃-N content in the C35 group was significantly higher than that in the C65 group (*p* < 0.05). However, the dietary concentrate-to-roughage ratio did not significantly affect volatile fatty acid concentrations in the rumen across all groups (*p* > 0.05).

**Table 3:** Effects of dietary concentrate to coarse ratio on rumen fermentation in yaks.

Items	Groups				*P*-value
	C80	C65	C50	C35	
pH	5.91 ± 0.18^a^	6.36 ± 0.12^b^	6.44 ± 0.13^b^	6.52 ± 0.12^b^	0.001
Acetate, mmol/L	0.31 ± 0.03	0.25 ± 0.05	0.31 ± 0.06	0.27 ± 0.05	0.215
Propionate, mmol/L	0.30 ± 0.07	0.29 ± 0.05	0.26 ± 0.07	0.24 ± 0.07	0.496
Isobutyrate, mmol/L	0.28 ± 0.06	0.27 ± 0.07	0.25 ± 0.06	0.26 ± 0.05	0.894
Butyrate, mmol/L	0.28 ± 0.06	0.26 ± 0.04	0.30 ± 0.07	0.25 ± 0.05	0.555
Valerate, mmol/L	0.28 ± 0.06	0.28 ± 0.05	0.30 ± 0.07	0.25 ± 0.06	0.994
Isovalerate, mmol/L	0.27 ± 0.06	0.26 ± 0.05	0.30 ± 0.06	0.26 ± 0.05	0.496
Caproic acid, mmol/L	0.27 ± 0.08	0.26 ± 0.05	0.29 ± 0.08	0.25 ± 0.07	0.881
Heptanoic acid, mmol/L	0.25 ± 0.06	0.24 ± 0.05	0.25 ± 0.06	0.29 ± 0.05	0.488
Total volatile fatty acid, mmol/L	2.24 ± 0.50	2.11 ± 0.41	2.26 ± 0.53	2.07 ± 0.45	0.635
NH3-N (μg/mL)	7.05 ± 1.24^ab^	5.50 ± 0.95^b^	6.92 ± 1.25^ab^	8.01 ± 1.31^a^	0.036
Cpr (mg/mL)	8.98 ± 0.89	13.64 ± 2.71	11.02 ± 1.34	11.00 ± 4.20	0.385

### 3.3 Macrogenomic analysis of the rumen of yak (*Bos grunniens*) on the ratio of dietary concentrates to coarser diets

Macrogenome sequencing generated a total of 289,847,786,688 clean reads, with 2,032,077,800 reads remaining after the removal of host genes. A total of 26,511,139 overlapping clusters were obtained following *de novo* assembly (Table 4). The rumen macrogenome consisted of 94.69% bacteria (799,538,812 reads), 0.18% eukaryotes (1,494,064 reads), 3.84% archaea (32,399,614 reads), and 1.29% viruses (10,893,934 reads).

**Table 4:** The quality of metagenome.

Sample	Clean reads	Optimized reads	Contigs	N50, bp	N90, bp	ORF	Total length, bp	Average length, bp
C80	13,903,260,367	92,332,750	824,054	1,160	384	1,237,253	672,278,571	543.36
C80	12,525,618,803	83,080,704	691,411	1,110	378	1,028,717	557,395,338	541.84
C80	12,473,630,269	82,797,730	720,355	1,226	390	1,087,615	606,463,284	557.61
C80	12,249,199,657	81,349,356	730,081	1,123	384	1,083,946	591,914,799	546.07
C80	11,816,538,350	85,646,160	899,471	976	372	1,300,229	678,693,567	521.98
C80	12,901,039,047	94,030,068	1,192,115	959	373	1,702,722	875,924,907	491.54
C65	12,600,763,034	83,641,608	1,091,306	1,007	375	1,580,782	836,396,382	529.1
C65	13,425,262,485	89,054,434	1,373,305	805	368	1,816,707	945,765,918	520.59
C65	13,546,272,704	89,878,068	1,042,273	1,008	375	1,497,918	801,032,382	534.76
C65	13,710,845,405	91,002,472	968,947	916	370	1,670,124	859,357,002	514.55
C65	14,206,545,633	94,421,684	955,907	976	371	1,373,035	717,693,138	522.71
C65	12,293,946,068	81,544,910	1,006,017	929	379	1,416,295	740,320,869	522.72
C50	12,063,669,993	80,049,636	1,349,988	742	358	1,790,625	871,357,341	486.62
C50	12,490,707,626	83,036,164	1,050,816	903	366	1,472,622	763,457,394	518.43
C50	13,017,401,372	86,405,862	1,559,290	716	358	2,066,892	960,579,855	464.75
C50	11,379,444,284	75,537,246	1,268,718	759	360	1,683,794	827,657,613	491.54
C50	11,482,116,100	76,183,884	1,086,630	904	376	1,542,543	789,644,889	511.91
C50	15,000,903,467	99,902,780	1,400,922	806	363	1,935,251	927,013,605	479.01
C35	11,748,619,245	77,939,614	1,230,899	690	353	1,593,854	757,361,574	475.18
C35	11,816,538,350	78,400,206	1,332,910	770	361	1,770,954	876,824,070	495.11
C35	11,818,459,540	78,420,622	1,187,973	723	356	1,556,368	754,435,938	484.74
C35	12,808,669,262	85,055,266	1,207,128	865	369	1,670,124	859,357,002	514.55
C35	12,209,137,695	80,994,168	1,223,643	811	366	1,659,905	840,138,456	506.14
C35	12,262,458,299	81,372,408	1,116,980	757	361	1,487,622	732,036,984	492.09

ORF, open reading frame.

**Figure 1:**
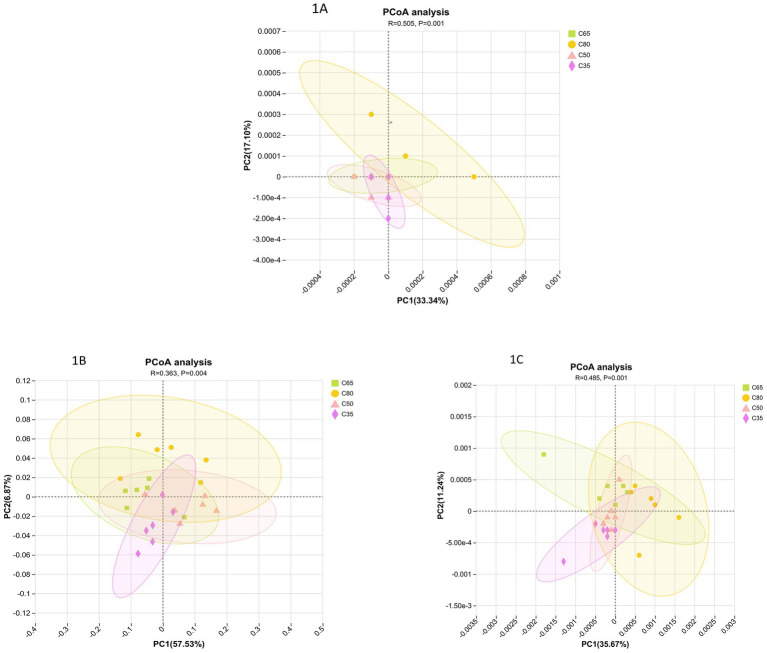
Principal coordinates analysis (PCoA) profile of microbial diversity based on Bray–Curtis distance matrixes. **(A)** Bacteria; **(B)** Fungi; **(C)** Archaea.

Principal Coordinate Analysis (PCoA) revealed distinct separation between all four dietary concentrate-to-roughage ratios based on bacterial, fungal, and archaeal species (Figure 1). In the PCoA analysis of bacterial communities, significant intergroup differences (*p* < 0.05) with a medium effect size (*R* = 0.509) were observed across dietary groups. The separation between high and low concentrate groups was most pronounced along the PC1 axis, indicating that dietary concentrate-to-roughage ratio significantly influenced bacterial community structure. In the PCoA of the fungal community, the difference between groups was significant (*p* = 0.004) but weak (*R* = 0.363), suggesting that the fungal community was less sensitive to dietary changes. Similarly, PCoA of the archaeal community also showed significant differences between groups (*p* < 0.05, *R* = 0.485).

### 3.4 Analysis of rumen species and functional composition in yaks by dietary concentrate ratio

**Figure 2:**
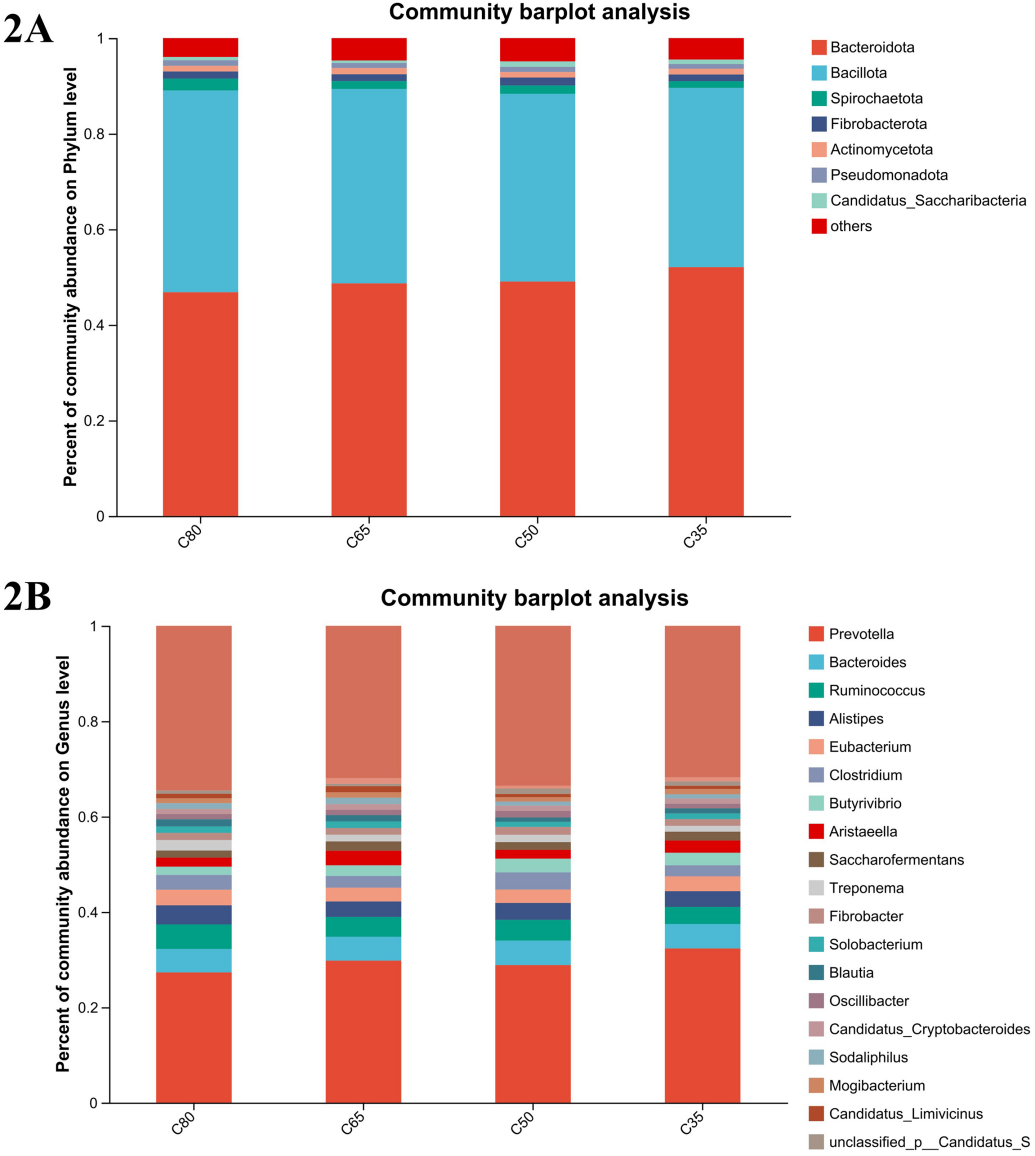
Bacterial composition of rumen samples from yaks with different concentrate and roughage ratios. Bacterial composition at phylum **(A)** and genus **(B)** level.

In all groups, a total of 10phyla, 225classes, 406orders, 722families, and 4,804genera were identified. Based on NR annotation results, the relative abundance of dominant taxa at the phylum and genus levels is shown in Figures 2A,B. At the phylum level, the most abundant phyla were Bacteroidota and Bacillota, accounting for 46.13, 43.7, 45.99, and 49.6% of the total reads in the four experimental groups for Bacteroidota, and 38.55, 39.41, 36.82, and 35.74% for Bacillota, respectively (Figure 2A). Bacteroidota were particularly enriched in the C35 group, with their relative abundance significantly lower in the C80 group compared to the C35 group (*p* < 0.05). Additionally, Bacillota was more abundant in the C80 group, followed by Euryarchaeota (3.51%), Spirochaetota (2.3%), Uroviricota (1.64%), Fibrobacterota (1.4%), Actinomycetota (1.1%), Pseudomonadota (1.08%), and Candidatus_Saccharibacteria (0.63%).

At the genus level, the dominant genera were Prevotella (Prevotella spp.), Bacteroides (Bacteroides spp.), and Ruminococcus (Ruminococcus spp.), with Ruminococcus being the most abundant and significantly enriched in the C80 group (*p* < 0.05). Prevotella was significantly more abundant in the C35 group than in the C80 group (*p* < 0.05). Clostridium was significantly more abundant in the C50 group compared to the other three groups. Other genera with lower abundance included unclassified Caudoviricetes (0.97%), unclassified Candidatus_Saccharibacteria (0.89%), Sodaliphilus (0.85%), and Candidatus_Limivicinus (0.63%) (Figure 2B).

### 3.5 Profile of dietary concentrate ratio on rumen microbial function in yak (*Bos grunniens*)

**Figure 3:**
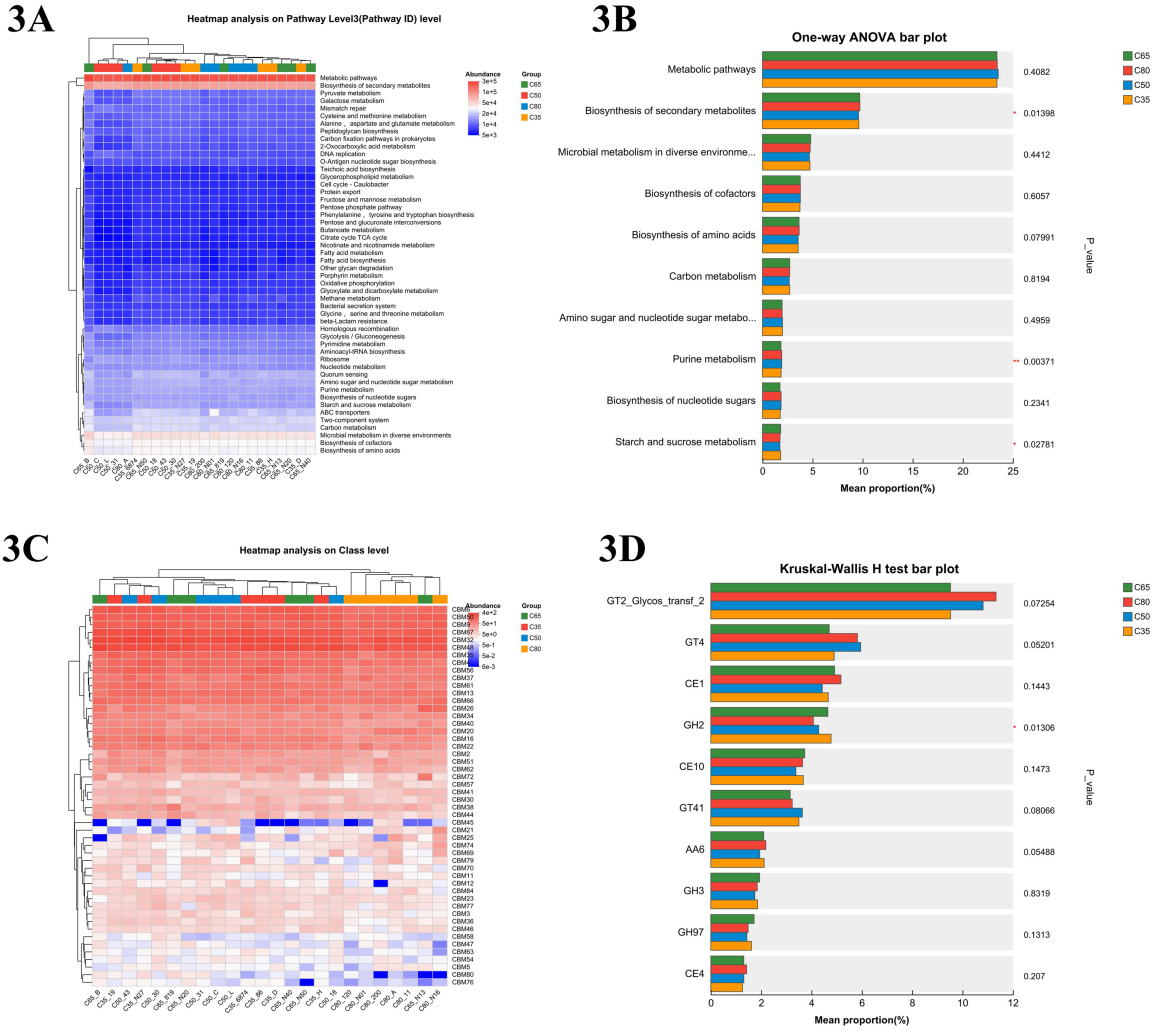
KEGG tertiary metabolic pathway maps and CAZy (carbohydrate-active enzyme) function maps for different dietary finishing and roughing ratios. **(A)** Functional heat map of KEGG tertiary pathway at different finishing ratios. **(B)** Relative abundance and statistically significant different of KEGG tertiary metabolic pathway in different finishing ratio groups by one-way ANOVA (One-way ANOVA) histogram. **(C)** Heat map of CAZy (carbohydrate-active enzyme) function. **(D)** Relative abundance and statistical differences of Kruskal–Wallis *H*-test histograms on CAZy (carbohydrate-active enzyme) family in different dietary groups.

To investigate the effects of different dietary concentrate-to-forage ratios on rumen microbial function in yaks, microbial community functional pathways were predicted using the Kyoto Encyclopedia of Genes and Genomes (KEGG) database (Figure 3A). The heatmap analysis revealed that microbial communities across all dietary groups were primarily enriched in core metabolic pathways, including carbon metabolism, amino acid biosynthesis, and nucleotide metabolism. Additionally, oxidative phosphorylation, glycolysis/gluconeogenesis, and fatty acid biosynthesis exhibited high levels of abundance. In the C35 group, microbial functions were particularly enriched in starch and sucrose metabolism, as well as fructose and mannose metabolism, suggesting enhanced carbohydrate utilization. In contrast, the C80 group showed higher expression levels of pathways related to cellulose degradation and methane metabolism. As shown in Figure 3B, different dietary treatments had significant effects on secondary metabolite biosynthesis (*p* = 0.007261) and purine metabolism (*p* = 0.003893). However, other core metabolic pathways, such as carbon metabolism and amino acid biosynthesis, remained relatively stable across dietary groups (*p* > 0.05).

The CAZy-level functional heatmap (Figure 3C) revealed significant differences (*p* < 0.05) in the expression abundance of carbohydrate-binding modules (CBM), glycoside hydrolases (GH), glycosyltransferases (GT), and other CAZy families among the dietary groups. Notably, CBM48, CBM20, and CBM13 were more highly expressed in the C35 group compared to the other groups, suggesting enhanced microbial capacity for binding and utilizing starch and related polysaccharides under lower concentrate conditions. In contrast, CBM50 and CBM6 exhibited higher relative abundance in the C80 group, which may be associated with increased activity in peptidoglycan and cellulose recognition. The C65 group showed a balanced expression of multiple CBM families, including CBM4 and CBM32, which are involved in both soluble and insoluble carbohydrate interactions, potentially contributing to the superior feed efficiency observed in this group. To further quantify these differences, the Kruskal–Wallis *H* test was applied to explore functional pathway variations between the groups, as shown in the histograms (Figure 3D). The abundance of GH2 (glycoside hydrolase 2) was significantly different between the dietary groups (*p* = 0.01306), whereas the expression of other enzymes, The C35 group exhibited higher expression of GH2, while the C80 group showed a higher abundance of enzymes like GT2 (Glycos_transf_2), AA6, and CE1. Additionally, the C35 group had the highest abundance of GH2.

### 3.6 Analysis of rumen microbial LEfSe microbial community and KEGG metabolic pathway in yak by dietary concentrate ratio

**Figure 4:**
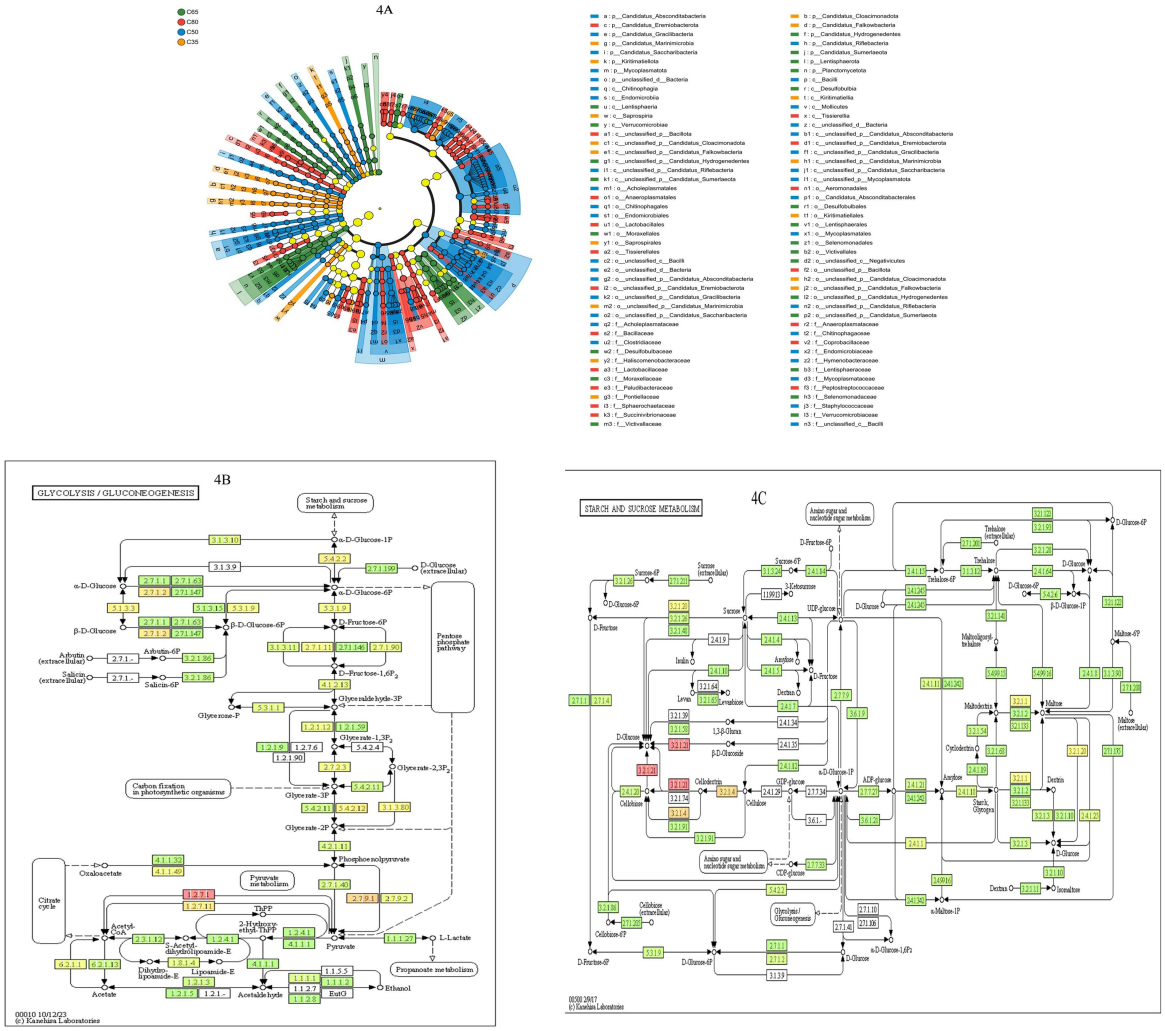
The effects of dietary concentration ratio on the LEfSe microbial communities and KEGG metabolic pathways in the rumen of yaks. **(A)** Based on the NR annotated (Domain-Spices) LEfSe species hierarchical map, the nodes of different colors in the figure indicate the microbial taxa that were significantly enriched in the corresponding groups and had a significant effect on the differences between the groups, while the nodes of yellowish color indicate the microbial taxa that did not have a significant difference in the different subgroups or did not have a significant effect on the differences between the groups. The yellowish nodes indicate microbial taxa with no significant difference in different groups, or no significant effect on intergroup differences. **(B)** KEGG metabolic pathway (glycolysis/glycolysis pathway ko00010), where each box with a filled color represents a sample or group of samples, and the shade of the color represents the change in abundance of the enzyme in different samples or subgroups. **(C)** KEGG metabolic pathway (starch and sucrose metabolic pathway ko00500).

Based on LEfSe hierarchical tree diagram analysis and KEGG metabolic pathways (glycolysis pathway ko00010 + starch and sucrose metabolism pathway ko00500), we further investigated the effects of the dietary concentrate-to-forage ratios on rumen microbial composition, sugar metabolism pathways, and methanogenesis. The LEfSe analysis (Figure 4A: among them, orange nodes: C35-enriched taxa; blue nodes: C50-enriched taxa; red nodes: C65-enriched taxa; green nodes: C80-enriched taxa) indicated that the C35 group was significantly enriched in fiber-degrading bacteria (Fibrobacter, Ruminococcus, Butyrivibrio), which predominantly degrade cellulose and hemicellulose, promoting the expression of cellulase (EC: 3.2.1.4) and β-glucosidase (EC: 3.2.1.21) (LDA score > 3.5, *p* < 0.05). In contrast, the C80 group was significantly enriched with sugar-fermenting bacteria (Prevotella, Bacteroides, Streptococcus), which primarily degrade starch and sucrose via *α*-amylase (EC: 3.2.1.1) and maltase (EC: 3.2.1.20) (LDA score > 4.0, *p* < 0.01). Additionally, the relative abundance of methanogenic bacteria (Methanobacterium, Methanosarcina) was significantly higher in the C80 group (*p* < 0.01). KEGG metabolic pathway analysis (Figures 4B,C) revealed that, in the C35 group, cellulase (EC: 3.2.1.4) and β-glucosidase (EC: 3.2.1.21) activities were significantly increased (*p* < 0.05), enhancing cellulose degradation and resulting in the release of glucose. Glucose then entered the glycolytic pathway (ko00010) at a relatively low rate, with its metabolites predominantly being acetic acid (Acetate) and propionate (Propionate) (*p* < 0.01). In the C80 group, rapid glucose metabolism was observed, with Prevotella and Bacteroides promoting the rapid degradation of starch and sucrose. The expression of α-amylase (EC: 3.2.1.1) and maltase (EC: 3.2.1.20) was significantly elevated (*p* < 0.01), accelerating glucose metabolism. Moreover, the activities of hexokinase (EC: 2.7.1.1) and fructose phosphokinase (EC: 2.7.1.11) were increased in the glycolytic pathway (ko00010) (*p* < 0.001).

## 4 Discussion

The feeding behavior of animals is fundamental to their nutrient intake, which drives growth and development. As the ratio of concentrate to roughage in the diet decreases, intake of dry matter (DM), organic matter (OM), crude protein (CP), and ether extract (EE) increases, while intake of neutral detergent fiber (NDF) decreases. This is primarily due to the higher content of soluble nutrients and lower fiber content, which also explains the improved digestibility of DM and OM (Barbosa et al., 2017). In ruminant feeding, balancing energy and protein is crucial. In this study, the diet of the C65 group provided a high level of energy while maintaining sufficient crude protein, facilitating efficient bacterial protein synthesis by rumen microorganisms. Conversely, the C35 group exhibited lower total weight gain, likely due to the low concentrate proportion and inadequate energy supply.

Interestingly, despite the high energy intake in the C80 group, no additional growth advantage was observed, likely because, beyond a certain threshold, excess energy intake does not translate into higher growth rates. Instead, it may be used for fat deposition or dissipated due to increased metabolic load (Marino et al., 2006). The present study also found that body slant length was significantly greater in the C65 group compared to the C50 and C35 groups, suggesting that a moderate concentrate level contributes to both bone growth and muscle deposition. High-concentrate diets typically promote the synthesis of propionic acid, which directly enters the gluconeogenesis pathway, providing energy for muscle growth (Hocquette and Bauchart, 1999). Chen et al. (2015) observed that increasing the dietary concentrate-to-forage ratios from 30:70 to 50:50 positively impacted housed yak growth performance, with higher dry matter intake and average daily gain (ADG) in yaks fed lower concentrate diets compared to those on higher concentrate diets, which aligns with the results of the present study. Similarly, Ku et al. (2021) found that increasing the concentrate-to-forage ratios significantly increased the total calf gain and final slaughter weight in calves fed high-concentrate diets, which is consistent with the present experimental results. However, the ADG of yaks in group C65 was the highest compared with the other three groups, possibly due to the unique characteristics of yaks. To adapt to the plateau environment, yaks are more tolerant of roughage, and increasing the concentrate ratio may predispose them to subacute rumen acidosis. Therefore, an appropriate concentrate-to-roughage ratio is essential for promoting yak feeding, enhancing ADG, and achieving better fattening outcomes.

The rumen is a unique digestive organ in ruminants, and maintaining a stable internal environment within the rumen is crucial for their overall health. Key indicators such as pH, ammonia-nitrogen (NH3-N), and volatile fatty acids (VFAs) are essential for monitoring the stability of the rumen environment and reflecting its fermentation status (Tomczak et al., 2019). Generally, the optimal pH range for microbial growth and reproduction in the rumen is between 6.2 and 7.0. When the rumen pH falls below 6.0, it can lead to subacute rumen acidosis (SARA), which inhibits the activity of fiber-degrading bacteria (Ruminococcus and Fibrobacter), thereby reducing crude fiber digestibility and exacerbating rumen acidosis (Grilli et al., 2016). In the present study, the pH values of the C65, C50, and C35 groups ranged from 6.36 to 6.52, while the pH of the C80 group fell below 6.0. This suggests that a certain level of concentrate in the diet can induce rumen acidosis, particularly when the concentrate-to-forage ratios reaches 80:20. As the proportion of concentrate in the diet increased, rumen pH decreased significantly, a finding consistent with previous studies by Khafipour and Grilli (Khafipour et al., 2016; Grilli et al., 2016). This decrease in pH is likely due to the high concentrate level in the C80 group, which, when combined with high-roughage feeds, stimulated rumen microbial fermentation and increased VFA production.

Rumen endogenous nitrogen (NH3-N) is the end product of the breakdown of feed and non-protein nitrogen, and plays a crucial role in rumen microbial protein synthesis (Lv et al., 2020). Llamas-Lamas et al. (1991), in a study involving three diets with concentrate-to-roughage ratios of 15:85, 30:70, and 45:55, found that as the dietary concentrate-to-forage ratios increased, rumen NH3-N concentration decreased significantly, while microbial protein (MCP) concentration tended to increase. This suggests that the concentrate-to-forage ratios can influence rumen NH3-N levels, which in turn may affect microbial protein synthesis. In the present study, rumen NH3-N concentration decreased with increasing dietary concentrate levels, but it rose in the C80 group. Similarly, microbial protein (MCP) concentration increased with higher concentrate levels, yet showed a decline in the C80 group. These changes may be attributed to the adjustment of the concentrate level. When the concentrate level increased, the rapid fermentation of rumen microorganisms effectively utilized the energy from non-structural carbohydrates, promoting NH3-N synthesis, which in turn accelerated microbial protein (MCP) production and reduced rumen NH3-N concentration. However, in the C80 group, the excessively high concentrate level likely disrupted the normal rumen fermentation process, disturbing the microbial community and interfering with protein synthesis. This disturbance was reflected by the increase in NH3-N concentration and the decrease in MCP concentration.

Volatile fatty acids (VFAs) in the diet are a primary energy source for the host, providing approximately 70 to 80% of the total energy and playing a critical role in supporting normal growth, reproduction, and other physiological functions (Li et al., 2016). Research has shown that alterations in the dietary concentrate-to-forage ratios can significantly influence the proportion of various VFA products, thereby modifying the fermentation pattern in the rumen (Polyorach et al., 2014). Maekawa et al. (2002) found that as the dietary concentrate ratio increased, the concentration of propionic acid in the rumen rose, while concentrations of butyric acid and total volatile fatty acids (TVFA) also increased significantly. Conversely, the concentration of acetic acid and the acetic acid/propionic acid ratio decreased markedly. Klevenhusen et al. (2013) reported similar findings, showing that with higher dietary concentrate levels, the concentrations of propionic acid, butyric acid, and TVFA gradually increased, while acetic acid concentration and the acetic acid/propionic acid ratio declined. These findings suggest that changes in the dietary concentrate-to-forage ratio can influence the composition of rumen fermentation products, likely by modulating the metabolic activities of rumen microbial communities. The present study observed a linear increase in total VFA, propionic acid, and butyric acid concentrations in the rumen as dietary concentrate levels increased, consistent with previous research (Polyorach et al., 2014). However, the concentration of acetic acid was lower in the C65 group compared to the C50 group, and conversely, it increased in the C80 group, which deviates from previous findings. This discrepancy may be due to the higher concentrate level in the C80 group, which could have altered rumen microbial growth and caused abnormal fermentation patterns. Furthermore, the shift from a 50:50 to a 65:35 concentrate-to-forage ratio resulted in an increase in rumen VFA content, whereas the 80:20 ratio had a detrimental effect. These results suggest that the 65:35 concentrate ratio is the most effective in yak diets.

This study analyzed the effects of diets with different concentrate-to-forage ratios on the rumen microbial communities in yaks using metagenomic sequencing. The aim was to explore the adaptive changes in rumen microbes and their potential mechanisms affecting host growth, nutrient digestion, and metabolic pathways. Principal Coordinate Analysis (PcoA) revealed that the bacterial community was most strongly influenced by the concentrate-to-forage ratios, which emerged as the primary factor for optimizing rumen function. Furthermore, the differences between bacterial and archaeal communities were relatively minor across the various concentrate-to-forage ratios, which aligns with findings from other studies (Hu et al., 2019; Islam et al., 2021). The present study showed that Firmicutes (thick-walled bacteria) and Bacteroidetes (bacillus-like bacteria) were the dominant phyla in the yak rumen microbial community, consistent with previous research (Bi et al., 2018). Firmicutes are primarily involved in the degradation of fibrous materials, while Bacteroidetes play a crucial role in breaking down non-fibrous materials (Evans et al., 2011). Both phyla are essential for maintaining the stability of the rumen microbiota and facilitating nutritional metabolism in ruminants.

Our findings indicated that the abundance of both Bacteriophage anomalies and Firmicutes was lower in the rumen of yaks in the C50 group compared to the C65 group, a result that somewhat deviates from previous studies (Reigstad and Kashyap, 2013). This difference may be attributed to the impact of the concentrate level on microbial substrate availability and the dynamics of microbial competition. In the C50 group, the lower concentrate level and insufficient fermentable sugars likely limited the growth of Firmicutes, while the higher fiber content may have failed to effectively promote the proliferation of Bacteriophage mimic. Conversely, the higher abundance of short-chain fatty acids (SCFAs) and the associated metabolic advantages in the C65 group likely facilitated the growth of these two major phyla, leading to a higher microbial abundance than in the C50 group.

The Firmicutes phylum was enriched in the C80 group, likely due to the increased substrate supply from the higher concentrate level and adaptive changes in the bacterial population. Firmicutes primarily utilize fermentable sugars and starch (Mao et al., 2013), and the high concentrate ratio in the C80 group provided a readily available source of carbon to promote their growth. Additionally, the higher concentrate level may have improved the efficiency of SCFA production, further enhancing the metabolic advantages of Firmicutes (Plaizier et al., 2021). Thus, the enrichment of Firmicutes in the C80 group is likely a result of the elevated substrate supply from the higher concentrate level, which provided them with a competitive advantage in the rumen microbial community.

In this study, KEGG database predictions indicated that microbial communities across all dietary groups were enriched in core metabolic pathways, including carbon metabolism, amino acid synthesis, and nucleotide metabolism, consistent with previous research (Cui et al., 2022). In the C35 group, microbial activity was particularly elevated in functional pathways related to glucose metabolism, as well as fructose and mannose metabolism. This may be attributed to the higher roughage ratio, which promoted the growth of anaplerotic phyla and other sugar-metabolizing microorganisms. In contrast, the C80 group showed higher expression levels in pathways associated with cellulose degradation and methane metabolism. This is likely due to the higher concentrate proportion in the C80 group, which increased the abundance of soluble carbohydrates, leading to rapid fermentation and the production of higher levels of lactic acid, propionic acid, and other fermentation products. This, in turn, stimulated the growth of cellulose-degrading bacteria, such as Clostridia and certain Firmicutes (Pang et al., 2022a,b). Regarding carbohydrate-binding modules (CBMs) in the carbohydrate-active enzyme (CAZy) database, CBM6, CBM50, CBM67, CBM32, and CBM48 were among the most abundantly expressed families across all treatment groups. Notably, the C80 group exhibited the highest relative abundance of CBM6 and CBM50, both of which are involved in cellulose and peptidoglycan binding, suggesting a potential enhancement of fiber-degrading and microbial cell-wall-targeting activity under high-concentrate feeding. Conversely, the C35 and C50 groups showed elevated expression of CBM13, CBM20, CBM26, and CBM61. These families are commonly associated with starch-binding and hemicellulose interaction, indicating that yaks fed lower-concentrate diets may rely more on microbes with enhanced affinity for plant polysaccharides. In particular, CBM20, a well-characterized starch-binding module, was more prominently expressed in the C35 group. The C65 group displayed an intermediate pattern, with moderate expression of both fiber- and starch-associated CBMs such as CBM32, CBM9, and CBM57. This balanced CBM expression profile may reflect a versatile carbohydrate-binding capacity of the rumen microbiota, enabling efficient degradation of both soluble and structural carbohydrates. This finding aligns with the superior sugar metabolism (ko00500 activity) and growth performance observed in the C65 group. Overall, these results suggest that dietary concentrate levels modulate the rumen microbial community’s CBM expression patterns, which may influence substrate specificity and carbohydrate utilization efficiency. This may be because the C35 and C50 diets, which provided more structural carbohydrates like cellulose and hemicellulose, promoted the increased abundance of polysaccharide-degrading CBMs (CBM6, which binds hemicellulose; CBM9, which binds β-glucan; CBM32, which binds complex polysaccharides; and CBM48, involved in starch degradation), to enable rumen microorganisms to efficiently utilize complex carbohydrates (Zhang et al., 2017). In contrast, the higher concentrate proportion in the C80 group, rich in easily fermentable carbohydrates, led to microorganisms preferentially degrading soluble sugars. This shift likely contributed to the reduced expression of CBM36, CBM77, and CBM3, which are primarily involved in cellulose binding (Zhang et al., 2018). Additionally, the high concentrate content may have inhibited fiber-degrading flora (Clostridium spp.), further decreasing the abundance of fiber-degrading CBMs (Wang et al., 2020).

LEfSe hierarchical tree diagram analysis and KEGG metabolic pathway analysis (including glycolysis pathway, ko00010, and starch and sucrose metabolism pathway, ko00500) revealed that dietary variations significantly influenced the diversity and structure of rumen microorganisms. In the C35 group, fiber-degrading bacteria such as Ruminococcus, Fibrobacter, and Butyrivibrio were predominantly enriched. These microorganisms are capable of producing key degrading enzymes, including cellulase and xylanase, which enhance the digestion of crude fiber in yaks (Pu et al., 2023). In contrast, the C80 group showed a significant enrichment of sugar-fermenting bacteria, including Prevotella, Bacteroides, and Streptococcus. These bacteria primarily catalyze the degradation of starch and sucrose through enzymes such as *α*-amylase (EC:3.2.1.1) and maltase (EC:3.2.1.20). This suggests that under high-concentrate dietary conditions, the metabolic function of the rumen microbial community shifts toward sugar fermentation, which may alter both the type of rumen fermentation and the energy supply patterns (Silva et al., 2025).

In addition, cellulase and β-glucosidase activities were significantly higher in the C35 group, indicating enhanced cellulose degradation by the rumen microorganisms in this group. This facilitated cellulose hydrolysis, leading to the release of glucose. Cellulase primarily catalyzes the breakdown of cellulose into cellobiose and short-chain oligosaccharides, while β-glucosidase further acts on these intermediates to release free glucose (Flint et al., 2012). Due to the high proportion of roughage in the C35 diet, the rumen microbial community was predominantly composed of fiber-degrading bacteria. As a result, the glycolytic pathway (ko00010) was relatively underactive, with acetic acid and propionic acid being the main fermentation products (Su et al., 2014). In contrast, the C80 group exhibited a significant shift toward rapid glycolysis, with a microbial community dominated by sugar-fermenting bacteria. *Prevotella* spp. degrade starch into maltose and dextrin by secreting α-amylase, while *Bacteroides* spp. express maltase to convert maltose into glucose, thus accelerating carbohydrate utilization (Martens et al., 2011). The glycolytic pathway (ko00010) was significantly enhanced in the C80 group, with marked increases in hexokinase and phosphofructokinase activities, indicating a faster rate of glucose metabolism. This accelerated glycolysis promotes the conversion of glucose to short-chain fatty acids (SCFAs), which provide energy to the host (Chen et al., 2022). The C65 and C50 groups exhibited distinct metabolic profiles in terms of sugar metabolism and fiber degradation. The C65 group showed more active sugar metabolism (ko00500) due to the higher concentrate proportion. Enzymes such as α-amylase (EC:3.2.1.1) and maltase (EC:3.2.1.20) were expressed at similar levels, promoting the breakdown of starch and soluble sugars. Additionally, the activities of hexose catabolism (EC:2.7.1.1) and fructose phosphate (EC:2.7.1.11) were enhanced, resulting in the rapid conversion of pyruvic acid to acetic acid and propionic acid, thus improving energy utilization efficiency. The C50 group maintained a dynamic balance between sugar metabolism and fiber degradation, with comparable cellulase (EC:3.2.1.4) and β-glucosidase (EC:3.2.1.21) activities. This group was enriched in fiber-degradation-associated CBMs (CBM6, CBM9, CBM32, CBM48), indicating an efficient breakdown of structural carbohydrates. As a result, the C65 group exhibited higher sugar metabolism, while the C50 group achieved a more balanced approach, ensuring a stable energy supply and replenishing the microbial community structure.

## 5 Conclusion

A comprehensive analysis revealed that the C65 diet was most effective in enhancing yak growth performance, optimizing rumen fermentation, and maintaining microbial balance. This diet not only ensured a sufficient energy supply but also facilitated the coordinated functioning of sugar metabolism and fiber degradation. It promoted the synthesis of short-chain fatty acids (SCFAs), improved feed conversion efficiency, and effectively maintained rumen microbial stability. In contrast, high-concentrate diets, while increasing the rate of sugar metabolism and enhancing the conversion of pyruvate to acetate and propionate, may lead to a decrease in rumen pH over time. This could inhibit the growth of fiber-degrading bacteria and stimulate the activity of methanogens, resulting in a significant increase in methane emissions, which poses potential environmental sustainability concerns. Although high-fiber diets help stabilize rumen pH and promote the expression of cellulose-degrading enzymes, they may limit the growth potential and production performance of yaks due to insufficient energy supply. Therefore, in yak farming, optimizing the concentrate-to-forage ratios is crucial. This strategy should be tailored to meet production goals, enhance environmental sustainability, and minimize feed costs, thereby balancing growth benefits with ecological impacts to achieve a sustainable, high-efficiency, low-carbon farming model.

## Data Availability

The original contributions presented in the study are publicly available. This data can be found in here: Mendeley Data, Version 1, doi: 10.17632/d9tcf68x3c.1.

## Footnotes

^1^ www.illumina.com

^2^ www.majorbio.com

^3^ http://metagene.cb.k.u-tokyo.ac.jp/

^4^ http://www.ncbi.nlm.nih.gov/Taxonomy/taxonomyhome.html/index.cgi?chapter=tgencodes#SG1
